# State of the Art Bowel Management for Pediatric Colorectal Problems: Spinal Anomalies

**DOI:** 10.3390/children10091558

**Published:** 2023-09-15

**Authors:** Elizaveta Bokova, Ninad Prasade, John M. Rosen, Irene Isabel P. Lim, Marc A. Levitt, Rebecca M. Rentea

**Affiliations:** 1 Comprehensive Colorectal Center, Department of Surgery, Children’s Mercy Hospital, Kansas City, MO 64108, USA; 2 Division of Pediatric Gastroenterology, Hepatology, and Nutrition, Children’s Mercy Kansas City, Kansas City, MO 64108, USA; 3 Department of Pediatrics, University of Missouri-Kansas City, Kansas City, MO 64108, USA; 4 Department of Surgery, University of Missouri-Kansas City, Kansas City, MO 64108, USA; 5 Division of Colorectal and Pelvic Reconstruction, Children’s National Medical Center, Washington, DC 20010, USA

**Keywords:** bowel management, spina bifida, meningocele, spinal anomaly, tethered cord, constipation, fecal incontinence, enema, urinary incontinence, antegrade continence enema

## Abstract

Background: Patients with spinal abnormalities often struggle with fecal and/or urinary incontinence (up to 87 and 92%, respectively) and require a collaborative approach to bowel management in conjunction. Methods: To define existing approaches and propose state-of-the-art bowel management, a literature search was performed using Medline/PubMed, Google Scholar, Cochrane, and EMBASE databases and focusing on the manuscripts published July 2013 and July 2023. Results: Patients with spinal anomalies have impaired innervation of the rectum and anal canal, decreasing the success rate from laxatives and rectal enemas. Thus, transanal irrigations and antegrade flushes are widely utilized in this group of patients. Based on spinal MRI, the potential for bowel control in these children depends on age, type, and lesion level. On referral for bowel management, a contrast study is performed to assess colonic motility and evacuation of stool, followed by a series of abdominal X-rays to define colonic emptying and adjust the regimen. The options for management include laxatives, rectal enemas, transanal irrigations, antegrade flushes, and the creation of a stoma. Approximately 22–71% of patients achieve social continence dependent on the type and level of the lesion. Conclusion: Patients with spinal anomalies require a thorough assessment for continence potential and stool burden prior to initiation of bowel management. The optimal treatment option is defined according to the patient’s age, anatomy, and mobility. The likelihood of independent bowel regimen administration should be discussed with the patients and their caregivers.

## 1. Introduction

Spinal anomalies are congenital anomalies of neural development that occur in 1 out of 2000 live births and affect the brain, spine, or spinal cord in the first 4 weeks of gestation. Spina bifida (SB) is the most common diagnosis present in 1 out of 1500 births without anencephalia causing permanent disability and impacting neurologic function below the spinal cord defect [1]. Along with other spinal and sacral anomalies such as tethered cord, filum terminale, caudal regression syndrome, and sacrococcygeal teratomas, SB leads to the dysfunction of motor and sensory control of the gastrointestinal tract and urinary system resulting in the neurogenic bowel and bladder in as many as 92% and 87% of patients, respectively [2]. Of patients with spinal anomalies, up to 77% present with fecal incontinence [3,4,5,6], 59–73% struggle with urinary incontinence [4,5], and decreased quality of life [2,7] regardless of age, stool frequency, or amount of leakage [4].

A structured approach to bowel management in these patients can improve continence and allow them to achieve better functional outcomes [8,9,10,11,12,13,14]. Participation in a dedicated bowel management program (BMP) can help children with spinal anomalies to be continent in 77% of cases [15], decrease associated urinary concerns, and improve their quality of life [15,16].

A stepwise protocol for the evaluation and management of children with spinal anomalies helps achieve the desired long-term outcomes. The current review paper focuses on bowel management strategies for patients with spinal lesions as part of a manuscript series on BMP in children with colorectal diagnoses (anorectal malformations, Hirschsprung disease, functional constipation, and spinal anomalies) [17,18,19,20].

## 2. Methods

A review of the literature published before June 2023 in Medline/PubMed, Google Scholar, Cochrane, and EMBASE databases, including original studies, meta-analyses, randomized controlled trials, and systematic reviews, was performed focusing on manuscripts and books published over the last 5–10 years in English. Search keywords included: “bowel management”, “spina bifida”, “meningocele”, “spinal anomaly”, “tethered cord”, “constipation”, “fecal incontinence”, “enema”, “urinary incontinence”, “transanal irrigations”, and “antegrade continence enema”. The reference lists of the retrieved articles were checked for other relevant articles not found during the initial search. Manuscripts and book chapters providing novel insights or addressing current challenges in the field were prioritized. Eighty-four of the selected articles and book chapters were included in the current review. The data was reported in a narrative format focusing on the recent updates in the BMP for patients with spinal lesions and used to inform an in-depth, stepwise protocol for bowel management.

## 3. Factors Affecting Continence and Treatment

Bowel management in patients with spinal differences is influenced by a blend of neurological, anatomical, and practical factors. The neurogenic bowel, stemming from disrupted extrinsic nervous pathways, manifests as altered defecation dynamics in these patients. The location of the spinal lesion, coupled with individual mobility and broader socioeconomic contexts, further shapes their bowel management experiences. This paper offers an in-depth analysis of these multifaceted determinants.

### 3.1. Anatomic Factors and Age

Defecation control relies on coordination between bowel wall sensory and motor neuronal impulses for effective peristalsis [21]. There are two types of nervous systems regulating the passage of stool: (1) intrinsic enteric nervous system within the bowel wall, and (2) extrinsic system with sympathetic and parasympathetic nerves regulating the action of the intrinsic fibers [21]. Neurogenic bowel in patients with spinal lesions results from the impairment of the extrinsic nervous pathway while intrinsic nerves remain anatomically intact [22]. Children with spinal abnormalities tend to have poor sphincter relaxation, abnormal defecation dynamics with decreased sensation, and external sphincter squeeze effort [23,24,25,26,27].

**Figure 1:**
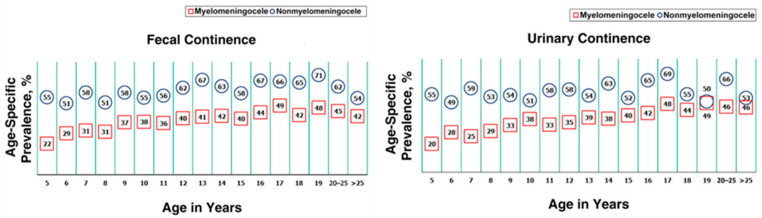
Fecal and urinary continence in patients with spinal anomalies depending on the type of lesion and the patient’s age. Numbers in squares reflect the age-specific prevalence of continence (fecal or urinary) in myelomeningocele patients, while those in circles pertain to non-myelomeningocele patients. Reprinted from Alabi, N.B.; Thibadeau, J.; Wiener, J.S.; Conklin, M.J.; Dias, M.S.; Sawin, K.J.; Valdez, R. Surgeries and Health Outcomes Among Patients with Spina Bifida. *Pediatrics* **2018**, *142*, 20173730 [30].

The spinal level of the lesion significantly affects long-term fecal and urinary continence [28,29] with sacral anomalies indicating a better prognosis than lumbar or thoracic lesions [28]. The type of anomaly also impacts functional outcomes. In the literature, myelomeningocele (MMC) and non-MMC (meningocele, lipomyelomeningocele, fatty filum) lesions are identified [30]. Patients with MMC tend to have a worse prognosis for fecal continence when compared to a non-MMC (22–49% vs. 51–72%, respectively) [3,28,30] and a lower rate of urinary continence (20–46% vs. 49–69%) [30]. Bladder control and bowel continence also depend on the patient’s age as shown in Figure 1 [30].

### 3.2. Mobility

Mobility is an important factor impacting the patient’s ability to administer their bowel regimen independently. Among adult patients with SB, up to 60% use wheelchairs [6,31], and 23–54% live independently [32] with the ability to self-care being one of the main goals of bowel management since childhood [33]. Of children 7–16 years of age, 60% are constantly dependent on their caregivers which negatively impacts their quality of life [34].

**Figure 2:**
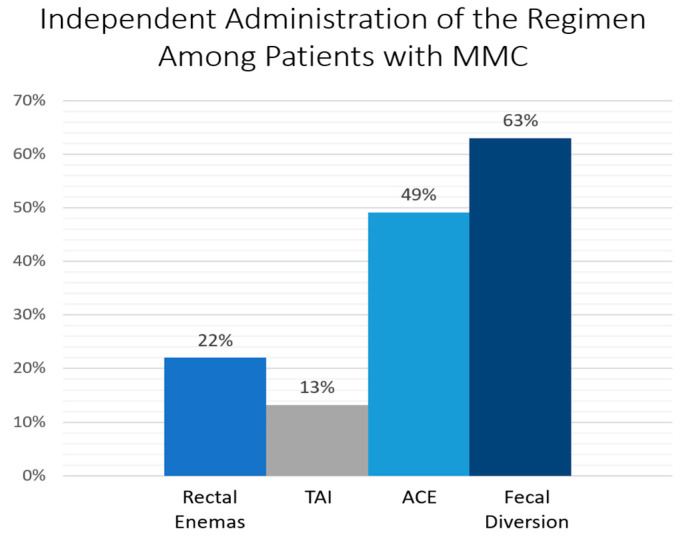
Independence from the bowel regimen in patients with myelomeningocele. ACE—antegrade continence enema; MMC—myelomeningocele; TAI—transanal irrigation. Data demonstrated from Kelly, M.S.; Wiener, J.S.; Liu, T.; Patel, P.; Castillo, H.; Castillo, J.; Dicianno, B.E.; Jasien, J.; Peterson, P.; Routh, J.C.; et al. Neurogenic Bowel Treatments and Continence Outcomes in Children and Adults with Myelomeningocele. *J. Pediatr. Rehabil. Med*. **2020**, *13*, 685–693 [35].

The time spent in the bathroom affects the patient’s compliance and their ability to independently use their bowel regimen. Patients using antegrade continence enemas (ACEs) have a twice higher risk of spending more than an hour in the bathroom to empty the colon when compared to those using transanal irrigations (TAIs) [34]. Due to the increased time required for bowel emptying with ACE flushes, and insufficient mobility of some patients with spinal lesions to administer rectal enemas or TAIs, fecal diversion might be the optimal treatment option taking much less time [22]. Kelly et al. reported the highest independence rates achieved with fecal diversion (63%), followed by ACE flushes (49%), rectal enemas (22%), and TAIs (13%) as demonstrated in Figure 2 [35].

## 4. Bowel Management Protocol

Before initiating bowel management in patients with spinal anomalies, the patient’s age, potential for fecal and urinary continence, desired goals of treatment, and mobility should be taken into consideration. Understanding these specifics and a strict follow-up with bowel regimen adjustment based on the abdominal X-rays allows us to determine the optimal bowel regimen, achieve higher adherence to treatment, and potentially better long-term outcomes [6,36,37,38].

**Figure 3:**
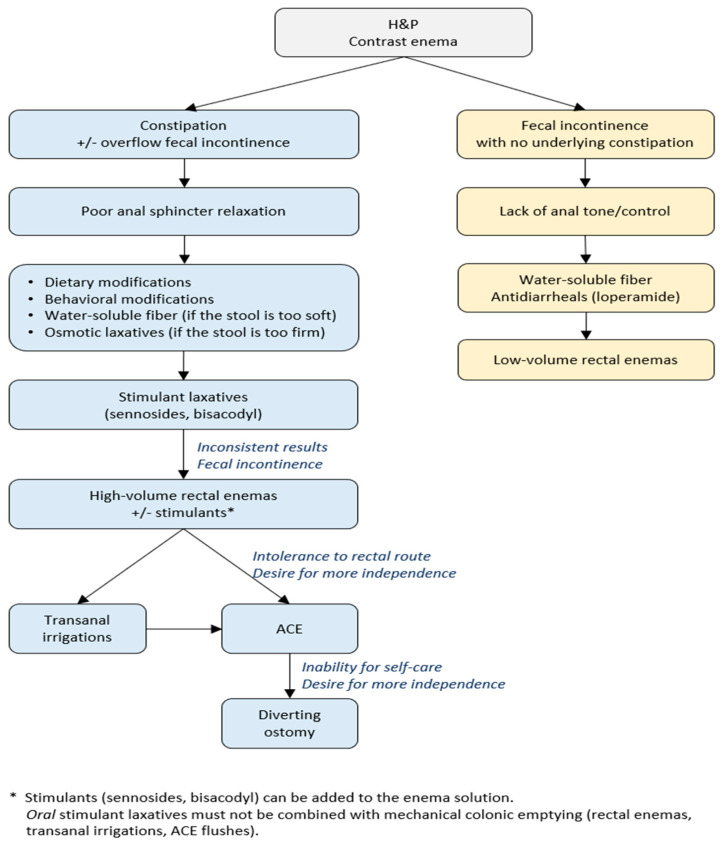
A stepwise protocol for bowel management in patients with spina bifida. ACE—antegrade continence enema; H&P—history and physical examination.

A stepwise algorithm of evaluation and bowel management in children with spinal lesions is demonstrated in Figure 3. The first evaluation step is obtaining the medical history, physical examination, and a contrast enema to assess the stool burden [20]. A nondilated colon on a contrast study is characteristic of patients with spinal anomalies and can be present in both hypo- and hypermotility cases [6]. In patients treated with an enema regimen, the colon can have a characteristic and yet unclassified pattern of colonic appearance with a dilated right colon and spastic left colon [39].

In patients with fecal incontinence, revealing the source of soiling is crucial to define the treatment strategy. Soiling can result either from constipation (overflow fecal incontinence) or poor anosphincteric control with or without hypercontractility. Children with fecal incontinence (without underlying constipation are managed with bulking agents such as water-soluble fiber and antidiarrheals (loperamide) with low-volume rectal enemas started if oral medications fail to improve bowel control.

Patients with spinal anomalies tend to have decreased colonic contractility and slow transit, with impaired motor and sensory function of the rectum and anal canal [15]. The goals of treatment in these children are to stimulate colonic motility and achieve optimal stool consistency with dietary and behavioral modifications, medical or mechanical treatment options, or a diverting ostomy.

### 4.1. Oral Medications

The type of medications used in children with spinal anomalies depends on the patient’s age, stool consistency, and time spent on the toilet to empty the bowel. Age-related concerns will be described in the next section of the manuscript.

The first line of medical treatment is bulking agents (water-soluble fiber) if the stool is too soft and difficult to control causing fecal incontinence. If defecation takes a significant amount of time and the stool is too hard to pass, osmotic laxatives (e.g., polyethylene glycol) or lubricants (mineral oil) can be used [40] to soften the stool and facilitate its passage alone or in addition to other treatment options (oral or mechanical regimen).

Stimulant laxatives such as sennosides increase colonic contractility and decrease transit time [40]. In the previous century, a concern about a possible carcinogenic effect of sennosides was raised due to the reported damage to the myenteric plexus based on rat studies [41,42]; however, this hypothesis has been rejected by more recent studies [43,44,45]. Another possible side effect of these medications precluding some physicians from using sennosides in the pediatric population is the risk of perineal blistering [46,47,48]. A recent study reported that the side effect occurs in patients with high doses of sennosides due to overstimulation of the colon and subsequent nighttime accidents which lead to irritation of the perineal skin [49]. This emphasizes a stepwise approach to sennosides dosing which decreases the rates of perineal blistering to 2% [49].

Lubiprostone has been reported as an effective adjustment to stimulant laxatives facilitating colonic motility by increasing intestinal fluid secretion [50]; however, in children with FC, this medication has not been shown to be effective when compared to the control group [51]. Further collection and analysis of objective data on the effectiveness of lubiprostone in SB patients are required.

There are no specific data on the combination of therapy which has led to a field of isolated clinical experts (usually in the nursing field) and a lack of published literature. The reality is that through trial and error and the experience of several bowel management strategies trialed, many children with spinal differences need to utilize more than one therapeutic option simultaneously to achieve results, but the outcomes to measure or standardization/escalation of therapy are lacking.

Theoretically, stimulant laxatives should not be combined with a mechanical regimen (rectal enemas, transanal irrigations, or antegrade flushes). After the mechanical solution cleans out the colon, laxatives lead to further contractions of the bowel resulting in leakage of stool. Osmotic laxatives can be used in addition to a mechanical regimen if the stool is too firm; however, there is no data on the outcomes of this combination in centers with a dedicated bowel management program. Children with inconsistent results on medical therapy or no response to laxatives are switched to mechanical treatment with rectal enemas.

### 4.2. Rectal Enemas and Transanal Irrigations

When compared to other groups of pediatric colorectal patients, children with spinal anomalies are more likely to be started on a mechanical regimen with rectal enemas [52]. Children who are unable to have predictable bowel movements with laxatives are also switched to rectal enemas. The goal of a mechanical regimen is to keep the patient clean for 24 h [15] with a large volume (300–500 mL) of saline and an additive (e.g., glycerin, Castile soap, bisacodyl) if needed [23,53]. Saline can be replaced with tap water only in patients older than 3 as in younger ones it can cause dehydration and hyponatremia [54]; however, there is no evidence supporting this practice. The solution is instilled for 5–10 min, and the patient is asked to hold it for 10 min and expel it within 30–45 min. Sodium phosphate-containing medications should be avoided due to the risk of rapid colonic fluid shifts with hyperphosphatemia, hypocalcemia, and hypokalemia [55,56]. A personalized solution/recipe is determined during the bowel management program (BMP) week and can be adjusted at further clinical visits and communication via email or other asynchronous technologies.

The major difficulty during enema administration in SB patients is leakage [15]. During enema administration the inability to completely squeeze the anal sphincters while trying to hold the enema solution is likely the reason. In this case, a greater volume can be introduced in the rectal balloon to decrease leakage during enema administration [15]. Passage of stool outside the enema administration results from overstimulation of the colon at the time of enema administration and indicates a potential need to decrease in the dose of stimulants. If an episode of fecal incontinence occurs long after the enema, insufficient colonic emptying may be the source of soiling, and the patient may require a higher dose of stimulants.

**Figure 4:**
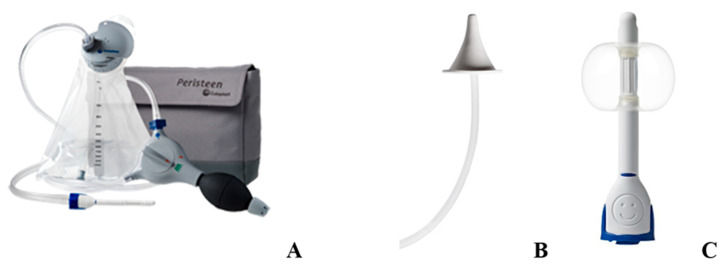
Transanal irrigation system (**A**) with a cone tip (**B**) or a catheter with a balloon (**C**).

Due to a decreased anal sphincter tone, patients with SB can fail laxative therapy [57,58] and be unable to hold enema solution [15]. In such cases, the child can be switched to TAIs. Irrigations are administered rectally using a cone tip at a younger age or a catheter with a balloon in patients over 6 years of age [57] (Figure 4) helping to retain the solution and avoid leakage.

TAIs have been reported to improve constipation, soiling, and quality of life in patients with SB [57,58,59]. At a 1.5-year follow-up, TAIs were effective in 86% of patients leading to a decrease in soiling and caregivers’ stress associated with the bowel regimen [59]. Given that the authors defined fecal continence as one or fewer soiling episodes per month, the rate of cleanliness for stool might have been higher if Rome IV criteria (one or fewer soiling episodes per week) were used [60,61].

Transitioning from a caregiver-dependent childhood to adulthood with the ability for self-care remains a major concern in patients with spinal lesions. The TAI system has a catheter with a balloon and a pump system to allow for the self-administration of retrograde enemas and, therefore, allows for some rate of independence [40]. Ausili et al. showed that TAIs had a lower frequency of soiling episodes, the time required for defecation, and the need for digital stimulation or evacuation when compared to their status on referral [62] thus increasing the patients’ independence from their bowel regimen. Even though TAIs effectively empty the colon in children with SB, their administration is associated with limited compliance [63] and only a 23% independence rate [35] which impairs the patient’s quality of life [34,35]. Given limited motility in children and adults with SB, TAIs may be a suboptimal treatment option, especially in older children aiming to achieve self-care (see Section 5 “Age-Related Bowel Management Concerns”).

### 4.3. Antegrade Continence Enemas

ACEs are administered via a channel between the bowel and abdominal wall. In comparison to rectal enemas and TAIs which allow retrograde regimen administration, ACE flushes empty the colon in a more physiologic way with the solution passing from the right colon to the distal portion of the bowel. Based on the intestinal part brought to the level of the skin, ACEs are divided into (1) a Malone appendicostomy, (2) Neomalone with a part of the colon used for the channel creation, and (3) cecostomy in which the flush is administered into the cecum.

In patients with spinal abnormalities, it is crucial to take the urologic plan of care into consideration as the appendix utilized for a Malone channel creation can be required for a Mitrofanoff channel creation. In cases when both an ACE access and urinary catheterize channel need to be created, a cecostomy can be placed with the appendix saved for a Mitrofanoff procedure. A cecostomy creation results in decreased anesthesia utilization and recovery time. Although, it can also be associated with a higher risk of leakage, wound infections, postoperative abdominal pain, and the need for additional interventions when compared to a Malone appendicostomy [64,65]; however, this statement is controversial [66]. As an alternative to a cecostomy, the appendix could be split based on its length with a detailed description of the technique covered in a related manuscript [17].

A recent manuscript by Kelly et al. showed that 40% of patients with MMC undergo a surgical intervention including an ACE procedure [35]. ACE flushes allow for a more “physiological” emptying of the colon in an antegrade manner starting from the most proximal part of the colon. In contrast, rectal enemas might not reach the right colon and, thus, lead to incomplete emptying and failure of bowel management. Some centers report the necessity of a successful rectal enema regimen before ACE creation [15]; however, some patients cannot tolerate a rectal route, do not benefit from rectal enemas, or desire more independence and should be considered candidates for an ACE creation.

Wiener et al. reported the highest rate of ACE use in adolescents [3]. ACEs provide the patients with a 4–5 times higher independence rate when compared to TAIs (56–63% vs. 13%, respectively) [35,58] resulting in an increased satisfaction rate [67].

Children with both bladder and bowel neurogenic dysfunction might require both mechanical emptying of the colon (ACE flushes) and urinary diversion (Mitrofanoff/Monti channel). The decision-making on the use of the appendix in these cases has been described in a related manuscript [17]. As an alternative to an ACE procedure in these patients, TAIs can be utilized for bowel management [68] and prevent the use of the appendix for a Malone procedure.

### 4.4. Diverting Ostomy

According to recent studies from the National Spina Bifida Patient Registry, 2–3% of patients with SB aged 5–19 years are managed with a diverting ostomy [3,35,69] with a higher prevalence in those with MMC when compared to the non-MMC group [69]. As the database included patients treated from 2006 to 2015, we suspect that the current rates might be lower since an ACE procedure and TAIs have been widely introduced into clinical practice.

The main advantage of fecal diversion is the significant reduction in the time needed to empty the bowel [22], avoidance of stool leaking on to the perineum and need for rectal therapy Even though in most cases, an ostomy is used as a “last resort” treatment option [70,71], the patients should be counseled about this treatment option earlier [72] as it can provide independence and ability for self-care [73,74].

## 5. Age-Related Bowel Management Concerns

**Figure 5:**
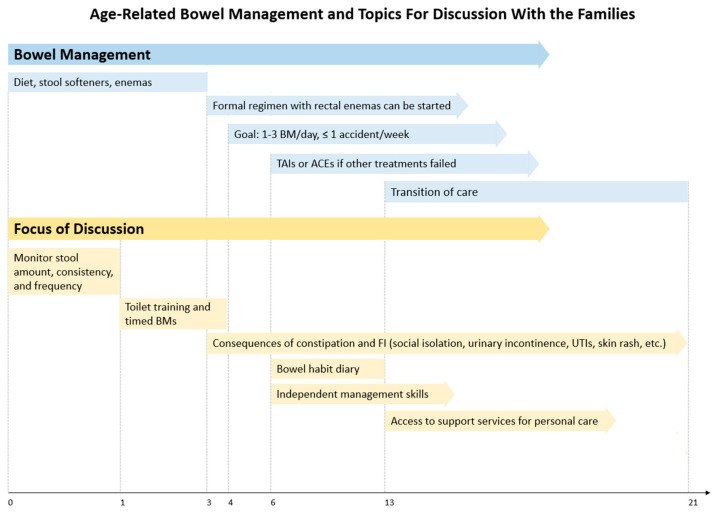
Age-related bowel management and topics for discussion with the families of patients with spinal anomalies. ACE—antegrade continence enema; BM—bowel movement; FI—fecal incontinence; OBGYN—obstetrics and gynecology; TAI—transanal irrigation; UTI—urinary tract infection. The scheme is based on the algorithms recommended by the Spina Bifida Association [75] and Stevens et al. [6].

Until recently, there was limited information on bowel management based on a child’s age. In the last few years, a few recommendations were published on this topic including a manuscript by Stevens et al. [6] and guidelines from the Spina Bifida Association [75]. The latter covered specific concerns for bowel management related to a child’s age which overlap in some patient groups and are demonstrated in Figure 5. The common concepts applicable to all patient groups not depicted in the scheme include moving from less to more invasive treatment options [75] and using skin barrier cream to manage skin rash if present [76]. An annual follow-up is required to adjust the bowel regimen if needed [6].

Importantly, a regimen that may work for the patient at one point in time as a child may not have the same results as the patient reaches adulthood which indicates changing the management strategy. For example, a child being clean on ACE flushes gains weight over time and develops ulcerations and/or cannot sit on the toilet long enough to empty the colon. In such a case, an end colostomy can be beneficial.

Around the age of 3, diet, osmotic laxatives, and rectal enemas are used to improve colonic emptying [6]. Dietary modifications should be considered before starting the child with medical treatment options [37], though that is notably difficult in the toddler age group. Breastfeeding in younger patients is preferred as it is easier to digest and allows for restoration of the microbiome of the bowel after surgery for a spinal lesion [75,77].

When the patient reaches the age of three to four, a formal bowel regimen with rectal enemas can be initiated [6]. Children typically have the developmental maturity required for toilet training, though this can vary. The goal of the treatment is for the child to have 1–3 bowel movements daily with one or fewer accidents per week [61]. Regimen adjustments are made, with escalation to TAIs or ACEs if other treatment options fail to improve continence. Collaboration with urology is started around the age of five with the assessment of the need for an ACE procedure with a simultaneous creation of a urinary catheterizable channel (Mitrofanoff/Monti) with or without bladder augmentation and bladder neck reconstruction [6].

Another aspect of bowel management is child milestones. In children younger than one, the caregivers are instructed to monitor the child’s stool consistency, frequency, and amount [8,78]. As the child grows, toilet (cleanliness for stool, dryness for urine) training becomes the goal to be achieved by the age of 4 (if developmentally appropriate) with timed voiding being one of the supportive techniques. From the age of three, families should be educated about the consequences of constipation and its effect on continence including social isolation, limited daily activities, shunt dysfunction, skin breakdown, and urinary concerns such as urinary tract infections, and urinary incontinence [34,37,62,79,80,81,82,83,84,85].

On reaching school age, the patients start seeking independence which should be encouraged with a bowel habit diary being a useful tool for the initiation of self-care [75]. The engagement of the school staff is vital to assist the child in the management of their health-related concerns [86]. In older children over 13 years, access to support services for personal care is ensured with the initiation of the transition of care to adult specialties which ideally should be completed by the age of 21 [87,88,89,90]. Other obstetric, gynecologic, and sexual concerns should be addressed at/after puberty as developmentally appropriate.

### Transition of Care

As mentioned in the previous section, a treatment option that is used to keep the child clean can become ineffective as the patient grows and reaches adulthood. Therefore, regular follow-ups are required to assess the stooling pattern and adjust the bowel regimen if needed.

Timely transition to adult specialties is vital for the achievement of the desired long-term outcomes and continuity of care. Among patients with SB, only 49% are transited to adult clinics [91]. Agrawal et al. reported that 34% of SB patients are transitioned to a multidisciplinary facility, while others continue to care in a pediatric multidisciplinary clinic (28%) or a regular adult clinic (34%) [89]. The barriers leading to low adherence to transition include health literacy [92,93,94], difficult coordination of care, and long wait times [91]. For further information on the transition of care to adult specialties, please refer to a related manuscript [17].

It is important to ensure that when reaching adulthood, the patients will receive multidisciplinary care [89] as among those transferred to adult specialties, 85% have urologic issues (urinary incontinence, urinary tract infections, difficulty with catheterization or urolithiasis) with 97% of these patients requiring some sort of surgical intervention [95].

## 6. Outcomes

**Figure 6:**
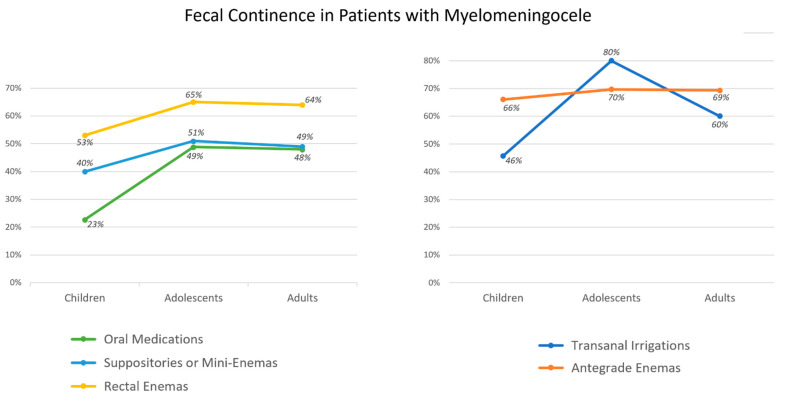
Fecal continence in patients with myelomeningocele based on their bowel regimen and age: young children (5–11 years), adolescents (12–19 years), and adults (20 years or older). Data from Kelly, M.S.; Wiener, J.S.; Liu, T.; Patel, P.; Castillo, H.; Castillo, J.; Dicianno, B.E.; Jasien, J.; Peterson, P.; Routh, J.C.; et al. Neurogenic Bowel Treatments and Continence Outcomes in Children and Adults with Myelomeningocele. *J. Pediatr. Rehabil. Med.* **2020**, *13*, 685–693 [35].

Clinical outcomes vary with age and treatment options. The continence rate was reported to increase with age for all bowel management options excluding TAIs, with the peak of continence in adolescence (Figure 6) [35]. In young children aged 5–11, ACE flushes are associated with the highest continence rate followed by large-volume rectal enemas (66% and 57%, respectively) [35]. In adolescents, TAIs lead to the best outcomes with an 80% success rate, while ACEs are effective in 70% of cases [35].

By eliminating concerns associated with defecation, bowel management also decreases the frequency of urinary tract infections [96], improves urodynamic characteristics [97], and urinary incontinence [98]. Compared to the group that managed for bladder dysfunction only, patients receiving both bladder and bowel management had 1.5 times the long dry period between clean intermittent catheterizations [98]. It is hard to report the exact data on bowel management outcomes in patients with SB due to the variability of fecal continence definition and age of the patients included in the studies [11,37,57,62,99,100].

## 7. Conclusions

Bowel management in children with spinal anomalies involves various strategies, from dietary changes and medications to surgical interventions. It is crucial to customize the regimen to the child’s specific needs, considering their mobility, age, and potential for fecal continence. By the time they reach toilet-training age, or earlier if fecal burden demands, structured approaches such as rectal enemas can be employed. To establish a consistent colonic emptying pattern, a transition to TAIs or ACE flushes may be necessary. Surgical options span from ACE placement to fecal diversion, with the latter being reserved for those unresponsive to other treatments. As they mature, the suitability of a chosen therapy may change, warranting periodic re-evaluations and necessary modifications to ensure consistent outcomes and foster caregiver ease and patient independence.

## Data Availability

No new data were created or analyzed in this study. Data sharing is not applicable to this article.
